# Using a Webcam to Assess Upper Extremity Proprioception: Experimental Validation and Application to Persons Post Stroke

**DOI:** 10.3390/s24237434

**Published:** 2024-11-21

**Authors:** Guillem Cornella-Barba, Andria J. Farrens, Christopher A. Johnson, Luis Garcia-Fernandez, Vicky Chan, David J. Reinkensmeyer

**Affiliations:** 1 Department of Mechanical and Aerospace Engineering, University of California Irvine, Irvine, CA 92697, USA; farrensa@uci.edu (A.J.F.); garciaf7@uci.edu (L.G.-F.); dreinken@uci.edu (D.J.R.); 2 Rancho Los Amigos National Rehabilitation Center, Rancho Research Institute, Downey, CA 90242, USA; johnson4@uci.edu; 3 Irvine Medical Center, Department of Rehabilitation Services, University of California, Orange, CA 92868, USA; vchan2@hs.uci.edu; 4 Department of Anatomy and Neurobiology, University of California Irvine, Irvine, CA 92697, USA

**Keywords:** proprioception, computer vision, pointing error, home-based rehabilitation

## Abstract

Many medical conditions impair proprioception but there are few easy-to-deploy technologies for assessing proprioceptive deficits. Here, we developed a method—called “OpenPoint”—to quantify upper extremity (UE) proprioception using only a webcam as the sensor. OpenPoint automates a classic neurological test: the ability of a person to use one hand to point to a finger on their other hand with vision obscured. Proprioception ability is quantified with pointing error in the frontal plane measured by a deep-learning-based, computer vision library (MediaPipe). In a first experiment with 40 unimpaired adults, pointing error significantly increased when we replaced the target hand with a fake hand, verifying that this task depends on the availability of proprioceptive information from the target hand, and that we can reliably detect this dependence with computer vision. In a second experiment, we quantified UE proprioceptive ability in 16 post-stroke participants. Individuals post stroke exhibited increased pointing error (*p* < 0.001) that was correlated with finger proprioceptive error measured with an independent, robotic assessment (r = 0.62, *p* = 0.02). These results validate a novel method to assess UE proprioception ability using affordable computer technology, which provides a potential means to democratize quantitative proprioception testing in clinical and telemedicine environments.

## 1. Introduction

Proprioception is our sense related to the perception of body position and movement [1,2,3]. Proprioception is important in both the control [4] and learning of movement [5,6]. Proprioceptive skill itself is also learnable, exhibiting similar properties to motor learning [7]. Consistent with this, skilled people, such as dancers [8,9], athletes [10], and pianists [11,12], have heightened proprioception. In rare conditions, when proprioception is completely lost, such as when genetic mutations affect mechanically sensitive ion channels in sensory neurons [13], the functional consequences are devasting and include a loss of walking ability, severely impaired balance, and a loss of dexterity [2]. A wide range of conditions are known to impair proprioception, including stroke [14,15,16], spinal cord injury [6,17], Parkinson’s disease [18,19], genetic conditions [20], orthopedic injuries [21], and neuropathies [22] including diabetic neuropathy [23,24].

Despite the prevalence and functional importance of proprioception deficits, they are rarely quantified during clinical examinations except in quick, coarse ways. For example, over one half of stroke survivors have upper extremity (UE) proprioception deficits [25]. However, a recent survey of 431 therapists found that only 1% reported assessing proprioception [26]. Tracking proprioceptive deficits could aid in targeting rehabilitation therapies. It could also potentially help in predicting responsiveness to rehabilitation therapy after stroke, as recent studies suggest that proprioceptive impairment is one of the most powerful predictors of treatment response [27,28]. This may be because proprioception serves as a “teaching signal” that guides motor learning and brain reorganization after stroke [29,30].

The motivation for this project was to leverage recent advances in computer vision to assess finger proprioception in a way that is more quantitative and precise than existing clinical approaches, while being easy, fast, and inexpensive. To do this, we were inspired by two common clinical approaches to assessing UE proprioception. In the first, a clinician asks the patient to close their eyes and try to move their fingertip from various locations to touch their nose with one finger [31,32]. In another, the clinician assesses the patient’s ability to localize their fingers by asking them to identify which finger is their right or left index, middle, ring, or pinkie finger [33,34]. Patients with proprioceptive impairment may overshoot or undershoot their movements, a phenomenon called dysmetria, or have difficulty identifying and localizing their fingertips [35]. While these assessments are quick and do not require equipment, their limitations include: poor resolution because they are scored in a binary success/failure manner, inconsistent results between different assessors, a lack of established normative benchmarks, and limited sensitivity [36,37].

To overcome these issues, previous apparatus-based proprioception assessments have been developed. A recent systematic review reports 1346 proprioception testing methodologies, many using specialized equipment [38]. One common approach focuses on finger localization tasks but uses commercial motion tracking systems to quantify errors. Such tasks have been used to quantify the effects of concurrent vision, static and dynamic movement, and aging on proprioception [31,39,40,41,42,43]. Another approach is to use robotic technologies to move the UE, asking participants to detect or reproduce features of the imposed movement [28,44,45,46]. While scientifically important, both motion capture-based and robot-based methods require expensive equipment that has limited portability and requires expertise to use. This has limited their deployment into routine clinical settings.

In the general area of sensorimotor assessment, an increasing number of studies are developing mobile phone-based applications with practicality and cost in mind [47,48]. A few applications have been developed for proprioception assessment, focusing on the knee. One example is iProprio [49], which uses headphones to instruct the participant to extend their knee to a desired angle, and the phone’s inertial sensors to measure the achieved angle, with an option to provide vibration feedback. MyProprioception [42], an iPhone application that uses smartphone photography analysis, was also used to measure the joint position sense of the knee by having an experimenter manually move the knee to an angle, then asking the subject to try to reproduce the angle and quantifying the error with the smartphone.

Similar app-based assessment tools using camera-vision have great potential for clinical and telehealth applications. Deep-learning-based motion capture techniques, such as OpenPose [50], MediaPipe [51], and Yolo-Pose [52], are a quantum leap in sensing human movement because they obviate the need for body markers [43] and provide real-time, robust, pose estimation. These techniques are increasingly being used in biomechanical analyses (e.g., [53]) and exercise apps (e.g., [54,55]), but we are not aware of their use in proprioception assessment. Here, we leveraged MediaPipe Hands, an open-source, real-time, on-device hand-tracking solution that is proficient in predicting hand landmarks to develop OpenPoint (v1.0.0), an easy-to-use, fast, affordable UE proprioceptive measurement system. This paper reports the design of OpenPoint as well as two experiments aimed at validating it, one with unimpaired participants and one with participants from a clinical population that frequently experience proprioception deficits: individuals who have experienced a stroke.

## 2. Materials and Methods

### 2.1. Design Overview

**Figure 1:**
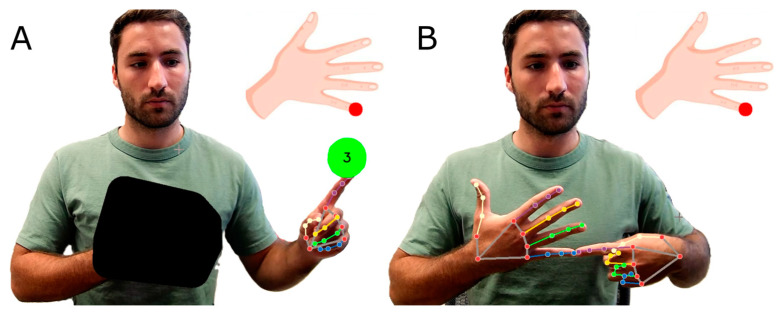
The visual display of the OpenPoint proprioception assessment, as implemented with a webcam. (**A**): The start position for the pointing task. Note that the image displayed to the participant is mirrored, so the user’s left hand appears on the left side of the screen. The assessment requires users to touch the fingertip of one hand with the fingertip of the other hand. The hand on the torso is the “target hand”, which is normally obscured using a graphically overlaid polygon, as shown on the left. (**B**): We removed the polygon to illustrate the accuracy of the finger tracking algorithm. The user is instructed to raise their pointing finger to a start target indicated by the green circle. The software then shows a target on the tip of one of the fingers of the cartoon hand (red circle). Following a three second countdown, the user is given an instruction to point and tries to touch the fingertip on their target hand, which is hidden by the polygon. Participants were instructed to refrain from directly looking at their own target hand. The tracking algorithm robustly tracks both fingertips and determines when the pointing finger stops moving, measuring the pointing error to assess proprioceptive ability.

The basic idea of the OpenPoint proprioception assessment is shown in Figure 1. The participant is instructed to hold a target hand in front of their chest (an achievable position even for a stroke-impaired arm), and then watch a mirror-image, live video of themselves on the computer screen. Positioning one hand in front of the chest ensures it remains outside the field of vision when participants are looking directly at the screen. The system can easily switch which hand is held in front of the chest as the target hand, but for simplicity we will describe it with the left hand as the target hand. We use computer vision to identify the location of the left hand and then block the view of this hand on the screen with an opaque polygon. On the remaining portion of the screen, we present instructions for the task: namely, to use the index finger of the right hand to touch a specific finger of the left hand, as indicated on a cartoon picture of a hand. A green start circle serves to standardize the start position for the pointing motion: individuals must first point to this green circle to trigger a “go” command (Figure 1).

OpenPoint uses computer vision to track the fingertips and to determine the pointing error projected onto the frontal plane. To identify when pointing has stopped, OpenPoint checks two conditions. First, the pointing index fingertip must be within a 15 cm radius of the target finger landmark, ensuring proximity to the target. Second, the standard deviation of the last 2 s (60 samples) of the array of pointing errors must drop below 5 cm, indicating that movement has slowed or stopped. By combining these criteria, OpenPoint approximates the moment when pointing has ceased. Participants were instructed to stop their movement following their initial pointing movement and were asked not to correct their movement.

### 2.2. Experimental Protocol

**Figure 2:**
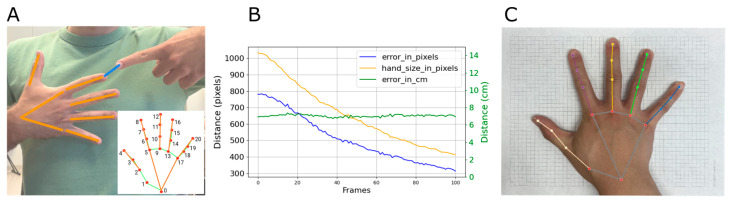
Pointing error calculation. (**A**) Example output from MediaPipe. The orange lines connect the landmarks returned by MediaPipe when the fingers are fully extended. We defined pointing error as the distance between the fingertips in the frontal plane (blue line). (**B**) Results from a simple experiment where the participants kept the distance between their fingers constant but moved their hands away from the camera by sliding backward on a rolling chair. The pixel-based pointing error (blue) decreased as the individual rolled back from the camera, as did apparent hand size, measured in pixels (orange line). The pixel-based pointing error (blue) has been multiplied by six to better show the decrease in distance. Dividing pixel-based pointing error by $han{dsize}_{px}$ produced a constant pointing error (green) that can be scaled to centimeters based on the calibration photos in (**C**). (**C**) An example calibration photo of participant’s hand lying on top of graph paper in order to calculate $han{dsize}_{cm}$.

We conducted two experiments with OpenPoint, one with unimpaired participants and one with people who had experienced a stroke. For both experiments, we fixed a webcam (Stylein HD 1080P Webcam, Guangzhou, China) to a small tripod in front of the participant, captured the trials with Python’s OpenCV library (4.10.0.84), and used MediaPipe (0.10.14) to track hand landmarks [51], allowing us to assess the normalized distance error between fingertips (Figure 2).

**Figure 3:**
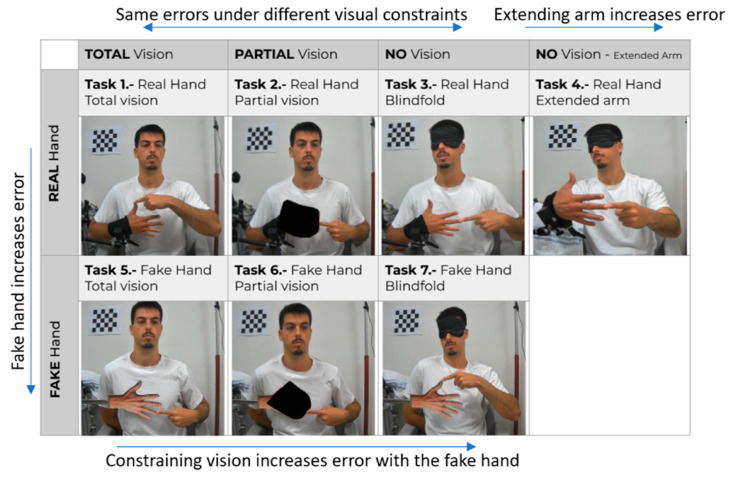
Graphical summary of the different tasks tested in Experiment 1.

1. Experiment 1—Unimpaired subjects:

22 young subjects (23.4 ± 4.1 years, 10 female, 20 right-handed), and 18 older subjects (62.3 ± 8.7 years, 12 female, 16 right-handed) with no neurological or visual deficits participated in Experiment 1. The participant sample size was selected based on the sample sizes used in similar studies to ensure the consistency and comparability of the results. This experiment was categorized as qualifying for UC Irvine’s Self-Determined Exemption for a brief, not physically invasive, behavioral intervention, and participants provided informed consent. Each participant from the young group performed a total of seven pointing tasks in the same order, from task 1 to task 7 (see Figure 3), whereas the older group performed the tasks in a randomized order.

The system can easily switch the hand that is held in front of the chest (target hand) to the right hand. However, for simplicity, all tasks are described with participants holding their left hand in front of their chest, since 90% of the subjects were right-handed. We designed the seven tasks to test finger localization accuracy under different visual and proprioceptive conditions. Tasks 1–3 tested the contribution of vision to the assessment, with conditions being the full vision (Task 1), with obscured vision of the target hand (Task 2), or blindfolded (Task 3). Task 4 tested the effect of proximal or distal positioning of the target hand on proprioceptive accuracy; a previous study that used a motion capture system found that extending the arm reduced proprioceptive accuracy [56] and we wanted to see if OpenPoint could replicate this finding. For Tasks 1–3, the participant’s wrist was held on an articulated arm support, with their hand positioned approximately 5 inches away from their torso. This setup was used to prevent participants from generating tactile inputs against their torso, which could be used to help judge hand location. For Task 4, participants extended their arm with their elbow straight, and the articulated arm supported both the upper arm and the wrist. Tasks 5–7 replicated the conditions of Tasks 1–3, except participants pointed to a fake hand (a cardboard cutout mounted on a stick) instead of their own hand. If successfully completing the pointing task requires proprioceptive information from the target hand, then the pointing error should increase in this condition, since no proprioceptive information is available from the target hand. Each task required the user to perform fifteen pointing movements to the target hand, three for each finger, which randomly changed from trial to trial. When queried, participants consistently reported no fatigue with repeated task performance in the experiment.

2. Experiment 2—stroke survivors:

Sixteen people (54.0 ± 12.8 years, 5 female, 15 right-handed, 9 right-side impaired) in the chronic phase post stroke (2381 ± 1499 days post stroke, 9 ischemic) participated in Experiment 2. The participants had varying levels of impairment, as assessed by the Upper Extremity Fugl-Meyer (UEFM) test [57]. The UEFM score evaluates UE motor impairment, and it ranges from 0 (complete paralysis) to 66 (normal movement). Participants were recruited from a stroke survivor database, the outpatient clinics at U.C. Irvine, as well as regional hospitals and stroke support groups. All visits took place at U.C. Irvine, and informed consent was obtained from all participants in accordance with a protocol approved by the UC Irvine Institutional Review Board.

**Figure 4:**
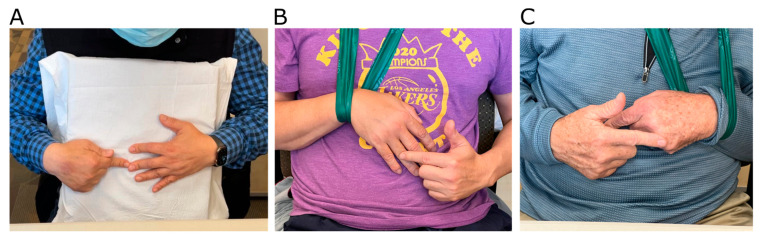
Examples of persons post stroke performing the pointing task. In Experiment 2, participants who had had a stroke sometimes could not extend the fingers of their target (hemiparetic) hand and were instructed to point to different landmarks on their hand depending on their capability. (**A**) Participant pointing to the fingertips while holding a foam pillow against the chest. (**B**) Participant pointing to the PIP joint while using an arm sling to hold his arm in a fixed position during the duration of the experiment. (**C**) Participant pointing to the MCP joint and using an arm sling.

Each stroke participant performed OpenPoint in a single-task condition, with their hemiparetic hand serving as the target hand and being positioned in front of their chest, which was obscured by the opaque polygon during pointing (Figure 3—Task 2). They performed thirty pointing movements with their unimpaired hand towards their impaired hand’s target landmarks. Participants who could fully extend their palm were instructed to point to their fingertips (Figure 4A). Those who could only partially extend their palm were instructed to point to the proximal interphalangeal (PIP) joint (Figure 4B). Participants who could not fully open their hand were instructed to point to the metacarpophalangeal (MCP) joint (Figure 4C).

For the participants with stroke, we initially asked them to replicate their target hand position we used for the unimpaired individuals (hand slightly separated from the torso) but found that they quickly experienced fatigue. So, we instructed them to rest their hands on a cushion or to use a sling. To prevent fatigue, participants were instructed to press a rectangular foam pillow against their chest (Figure 4A), which could help reduce errors in the sagittal direction. Participants who could not hold the pillow were allowed to use an arm sling (Figure 4B,C), which could provide torso tactile input on target hand location. However, this setup was not used for non-impaired individuals. We will discuss the difference in these conditions in Section 4.

### 2.3. Data Processing

MediaPipe Hand Tracking is a tool for robustly tracking hands using machine learning algorithms that relies on 2D images from a standard RGB camera. MediaPipe returns the 3D coordinates of 21 landmarks on the hand (see Figure 2A). MediaPipe infers depth from 2D images, which inherently lacks the direct measurement capability of depth sensors, such as those in Microsoft Kinect, Intel RealSense, or LiDAR sensors. Deep-learning approaches have shown a significant improvement in the performance of monocular depth estimation, but this topic is still evolving [58,59]. Therefore, we focused on frontal plane pointing errors (Figure 2A). We reasoned that proprioception errors should readily manifest in this plane even if they are 3D in nature.

MediaPipe returns the fingertip position as pixel coordinates, but we desired a measure of error in real-world coordinates. If the fingers are closer to the screen, the pixel-based pointing error between the fingertips will be larger compared to if the fingers are further away, even if the pointing error is constant (Figure 2B). Therefore, we developed a method to convert pointing error from pixels to centimeters using measured hand size. Specifically, we defined a variable called $handsize_{px}$ as the sum of the distances between the hand landmarks returned by MediaPipe. We took a photo of each stroke participant’s hand lying on a sheet of graph paper (Figure 2C) to calculate a constant called $han{dsize}_{cm}$, defined as the distances between the hand landmarks returned by MediaPipe, but this time in centimeters. To convert pixels to centimeters we used the perimeter of a page of US letter paper. Using these measurements, we calculated pointing error as:

(1) $$error_{cm}=\frac{han{dsize}_{cm}}{handsize_{px}}\times error_{px}$$

where MediaPipe data provides $handsize_{px}$ in real-time. Figure 2B shows the results of an experiment to demonstrate that this measure stays constant as the fingers vary in distance from the camera. Note that, throughout this paper, we will present the normalized pointing error between fingertips in cm and refer to it simply as “pointing error”. For participants who could not fully extend their fingers, we calculated their $handsize_{px}$ considering the distances to the knuckles instead of the fingertips, similarly to Figure 2A but choosing other landmark indices.

For the participants who had experienced a stroke, we acquired a picture of each participant’s hand on the graph paper (Figure 2C). For the unimpaired participants, we did not acquire the calibration pictures, so we used the average hand sizes obtained from the stroke participants on a sex-specific basis. $han{dsize}_{cm}$ was (49.87 ± 3.18) cm for women, and (54.84 ± 2.83) cm for men. The average $han{dsize}_{cm}$ of the fake hand used in tasks 5, 6, and 7 was always 60 cm.

### 2.4. Robotic Validation

**Figure 5:**
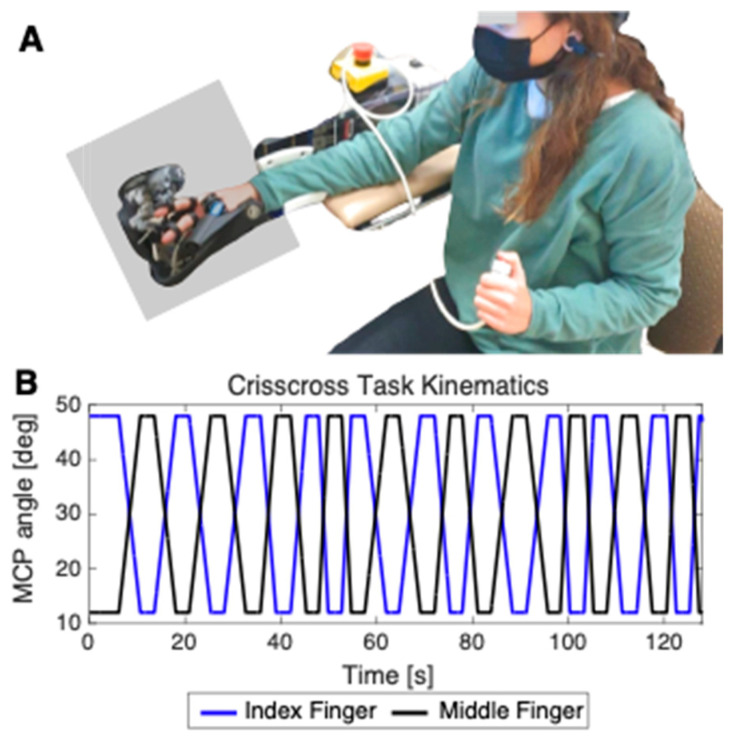
(**A**) Experimental setup for measuring finger proprioceptive error using the Crisscross assessments. For Crisscross, the FINGER robot moved the index and middle fingers in a crossing movement and participants were instructed to press a button with their other hand when they perceived them to be overlapped. The gray rectangle indicates the location of the opaque plastic divider used during the assessment to block the hand from view. (**B**) Example trajectories for the metacarpophalangeal (MCP) joint of the index (blue) and middle (black) fingers during Crisscross.

In order to test the concurrent validity of the OpenPoint assessment, we examined whether OpenPoint error correlated with finger proprioceptive error measured with a previously validated robotic proprioceptive assessment, the Crisscross assessment [27,28,44,60]. We implemented Crisscross with the FINGER robot (University of Idaho, Moscow, ID, USA), which uses two eight-bar mechanisms to independently move the index and middle finger. The Crisscross assessment used here comprised 20 finger-crossing movements, in which FINGER moved the index and middle fingers of the stroke-affected hand in opposing directions with the vision of the hand occluded. Participants were asked to indicate when the fingers crossed by pushing a button (Figure 5).

FINGER moved the fingers at identical speeds during each crossing, choosing randomly from the following set of speeds: {8, 8.5, 9, 10, 10.5, 11.5, 12.5, 14, 16, 18} deg/s. The range of movement was set to be between 15 and 45 degrees of flexion, such that all crossings occurred at 30 degrees flexion. Randomizing the crossing speeds ensured that the assessment was unpredictable. Crossing error was defined as the magnitude of the angular distance between the two MCP joints at the instant of button press. Each participant’s proprioceptive error was defined as the average crossing error across button presses.

The Crisscross assessment of finger proprioception was obtained as part of a clinical trial investigating the efficacy of gamified robotic hand therapy using the FINGER robot [28,45,46]. All participants completed the OpenPoint assessment after participating in this clinical trial, with the interval between the prior Crisscross assessment and the subsequent OpenPoint assessment being 30 ± 26 (mean ± SD) weeks.

### 2.5. Data Analysis

Despite the instruction to stop moving the finger after their initial pointing movement, some participants attempted to correct their pointing movements to perfectly align the fingertips. In such instances, we manually identified the frame prior to the movement correction.

Before analyzing pointing errors in the various conditions, we applied an outlier removal process using a criterion of two standard deviations from the group mean for all the pointing tasks in experiments 1 and 2.

To test the sensitivity of the OpenPoint assessment to different levels of visual information, the availability of proprioceptive information, and age, we performed a 3-way ANOVA on the pointing error measured in tasks 1–3 and 5–7 with fixed factors: vision (3 levels: full, partial, blindfolded), proprioception (2 levels: real hand, fake hand), and age (2 levels: young, older), and their interactions.

To test for the sensitivity in detecting the effects of target arm orientation and to observe the potential effects of the age groups, we performed a two-way ANOVA on pointing errors measured in tasks 3 and 4, with fixed factors: arm orientation (2 levels: close, far), age (2 levels: young, older), and their interaction. We used a two-way ANOVA instead of ANCOVA with age as a covariate because a preliminary analysis showed no linear relationship between continuous age and pointing error. Despite this, meaningful differences between categorical age groups (‘young’ and ‘older’) could still exist.

To determine the sensitivity to effects of stroke, we compared pointing errors measured for the stroke participants to unimpaired aged-matched adults using a one-way ANOVA with 2 levels (older and stroke). For all ANOVA analyses, we performed post hoc Tukey testing for significant main effects and their interactions. To cross validate OpenPoint’s proprioceptive measure with an independent measure, we performed a Pearson correlational analysis between OpenPoint error and finger proprioception error measured in the robotic Crisscross assessment using exclusively the stroke participants’ data.

## 3. Results

**Figure 6:**
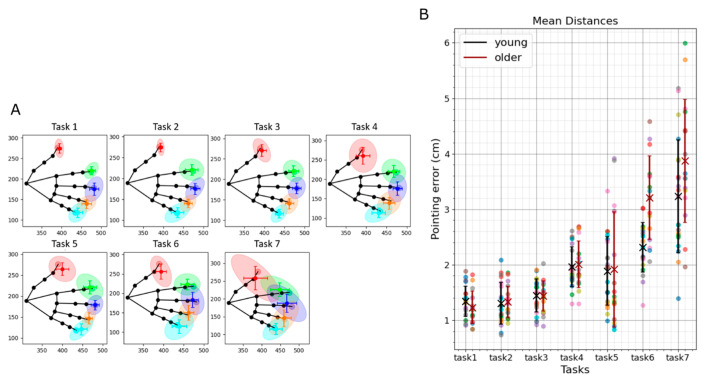
Experiment 1 results. In this experiment we evaluated the pointing error of unimpaired young (*n* = 22) and older (*n* = 18) individuals in different tasks. (**A**) Two-dimensional representation of the target hand (in black) showing the mean and standard deviation across participants of the pointing endpoint (in colors). The plotted data are from the young group. (**B**) Pointing error for each task (black: mean and SD for younger participants, dark red: mean and SD for older participants). Colored points show the pointing error for individual users.

We measured finger-to-finger pointing errors with OpenPoint in two experiments. Experiment 1 tested the sensitivity of OpenPoint error to three levels of visual information and two levels of availability of proprioceptive information (Figure 6). It also tested the effect of age and the position of the target hand (against the body or extended away from the body).

Experiment 2 tested the ability of the standard OpenPoint testing configuration (i.e., with partial vision) to assess proprioception ability in chronic stroke survivors. In this experiment, we also sought to validate OpenPoint error by comparing it with an independent measure of finger proprioception ability obtained with a robot assessment technique.

### 3.1. Experiment 1: Effects of Various Levels of Visual and Proprioceptive Information on Pointing Error

**Figure 7:**
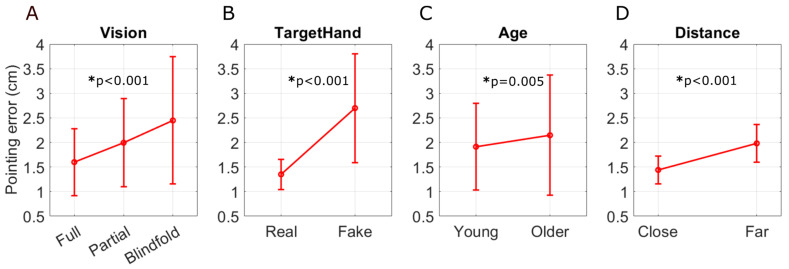
Pointing error as a function of different factors in Experiment 1. (**A**) Visual condition (ANOVA, *p* < 0.001). (**B**) Real or fake target hand (*p* < 0.001). (**C**) Age (*p* = 0.005). (**D**) Distance from the target hand to the body (*p* < 0.001). The error bars represent the standard deviation (SD) of the pointing errors.

The three levels of vision in Experiment 1 were (1) full vision of the torso and hands on the screen; (2) partial vision of the torso and hands on the screen, in which case the augmented reality polygon obscured the target hand; and (3) blindfolded. There was a significant main effect of visual condition (full, partial, and blindfolded, *p* < 0.001), proprioception (real vs. fake hand, *p* < 0.001), and age (young vs. older, *p* = 0.005) on OpenPoint proprioception error (Figure 7). The omnibus ANOVA also returned significant interactions between target hand and visual condition (*p* < 0.001), moderate interaction between visual condition and age (*p* = 0.05), and target hand and age (*p* = 0.002). Additionally, we found the main effects of arm orientation or distance (target hand close to the body vs. target hand extended out from the body, *p* < 0.001). We conducted further analyses to gain insights into each of these effects.

#### 3.1.1. Reducing Proprioceptive Information Increased Pointing Error

**Figure 8:**
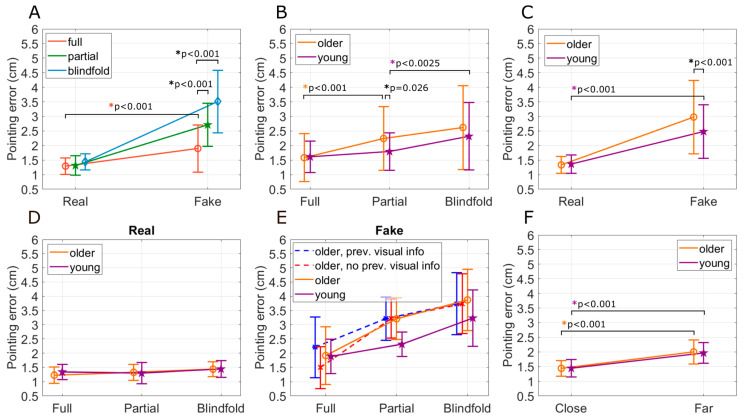
Further Analysis of Pointing Error from Experiment 1. (**A**) The effect of target hand conditions (real and fake) and visual condition (full, partial, and blindfolded), *p* < 0.001 (**B**) The effect of visual condition (full, partial, and blindfolded) and age (young and older), *p* = 0.05. (**C**) The effect of target hand (real and fake) and age (young and older), *p* = 0.002. (**D**) The effect of visual condition (full, partial, and blindfolded) and age (young and older) for the real hand, *p* = 0.59. (**E**) The effect of visual condition (full, partial, and blindfolded) and age (young and older) for the fake hand, *p* = 0.09, with additional lines showing the effects of task order. (**F**) The effect of distance (target hand close to the body vs. target hand extended out from the body) and age (older and young), *p* < 0.001. The error bars represent the standard deviation (SD) of the pointing errors.

Pointing to the fake hand caused significantly higher errors in all visual conditions compared to pointing to the real hand (Figure 8A, Tukey test, *p* < 0.001). That is, when we removed proprioceptive input from the target hand, pointing error significantly increased in all three visual conditions, although the increase was smallest for the full vision condition. Errors while blindfolded were significantly greater than the full- and partial-vision conditions (both *p* < 0.001). When pointing to the real hand, there were no significant effects of visual conditions (Figure 8A).

#### 3.1.2. Reducing Visual Information Increased Pointing Error

For both younger and older adults, pointing error increased as we removed visual information, going from full, to partial, to blindfolded conditions (Figure 8B), although this increase was marginally significant (i.e., there was a weak effect of visual information when considering both the fake and real hand conditions together, *p* = 0.05). However, because there is a strong significant interaction between vision and target hand (Figure 8A, *p* < 0.001) and target hand and age (Figure 8C, *p* = 0.002), we separated out the result for the real (Figure 8D) and fake hands (Figure 8E).

For pointing to the real hand, there were no significant main effects of age (*p* = 0.6) or visual conditions (*p* = 0.07) or interaction effects (*p* = 0.59). However, for pointing to the fake hand, there were main effects of age (*p* = 0.002). There was also a main effect of visual condition (*p* < 0.001), indicating that visual condition mattered more when proprioceptive information was not available from the target hand. However, we found no interaction effects (Figure 8E, *p* = 0.09) between visual condition and age, although the error increased with decreasing visual information.

#### 3.1.3. Effect of Task Order and Age

Younger and older individuals performed similarly on the real hand tasks, but the younger individuals performed better on the fake hand tasks (Figure 8C). But this might have been due not to age but because younger subjects always experienced the visual condition for this task first. Therefore, we tested whether the performance on the fake hand task was better for the older subjects if they experienced the visual condition first, since we randomized the tasks for them. The randomization procedure resulted in 61% of the older participants doing Task 5 before Task 6 and Task 7. Figure 8E shows that there were no significant differences in pointing error due to the task order (*p* = 0.282) (i.e., completing the ‘full’ condition before the ‘partial’ and ‘blindfold’) and thus receiving visual information earlier was no different to receiving it later. To further validate this, we combined the older data without segregating it by order and observed the main effects of age (*p* = 0.002) and vision (*p* < 0.001).

#### 3.1.4. Moving the Target Hand Farther from the Body Increased Proprioception Error

Task 3 (target hand close to the body) and Task 4 (target hand extended out from the body) were carried out with participants using their real hand as the target hand while being blindfolded. There was a significant main effect of target hand location (two-way ANOVA, close: 1.44 ± 0.28, far: 1.98 ± 0.38 [cm], *p* < 0.001), but no significant age-related effects (Figure 8F, *p* = 0.81). The two-way ANOVA confirmed that errors were driven by arm orientation rather than age, with no interaction effects (*p* = 0.77).

### 3.2. Experiment 2: Proprioceptive Pointing Error Was Increased After Stroke and Correlated with an Independent Assessment

**Figure 9:**
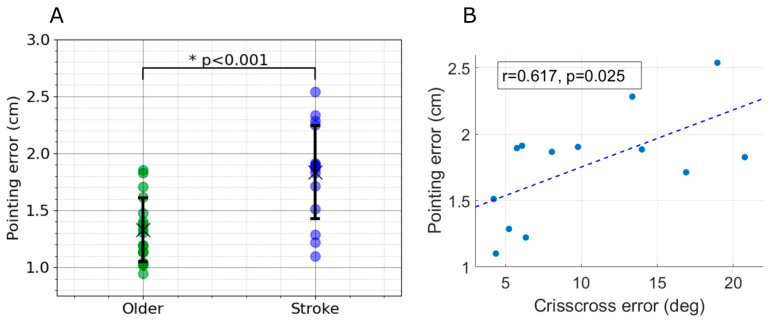
Results from Experiment 2. Proprioceptive pointing error was higher in persons who had experienced a stroke and was correlated with an independent, robot-based measure of their finger proprioception. (**A**) The pointing errors from Task 2 comparing the older and stroke groups. The stroke group had a significantly larger pointing error compared to the older group (*p* < 0.001). The error bars represent the standard deviation (SD) of the pointing errors. (**B**) OpenPoint pointing error was moderately correlated with the Crisscross finger proprioception error angular error. Each scatter point represents a participant.

In Experiment 2, participants with stroke performed the pointing task with their real hand as the target and with partial vision (i.e., with their target hand obscured by the augmented reality blob). This was equivalent to Task 2 from Experiment 1. Three participants were categorized as having severe motor impairment (UEFM < 30), five had moderate motor impairment (30 ≤ UEFM < 50), and eight had mild motor impairment (UEFM ≥ 50). We compared proprioceptive error between the stroke group and the age-matched older group using a one-way ANOVA and found a significant difference between groups (Figure 9A, *p* < 0.001). We observe that 53% of the stroke survivors exhibited mean pointing errors exceeding 2 standard deviations from the older group, while 27% had errors above 3 standard deviations. This indicates that the stroke group had significantly larger errors than the older group both on average, and in terms of individuals with mean pointing error outside of the normative range.

Finally, OpenPoint pointing error was moderately correlated with finger proprioception error measured robotically with the Crisscross assessment (Figure 9B, Pearson correlation, r = 0.617, *p* = 0.025) for those participants who were post-stroke.

## 4. Discussion

OpenPoint uses recent advances in computer vision to automate the quantification of pointing errors for a classic test of UE proprioception ability—finger-to-finger pointing with vision obscured—and requires only a standard webcam. The main results of the experimental validation were: First, we verified that this task depends on the availability of proprioceptive information from the target hand. This is because pointing error significantly increased when we instructed unimpaired participants to point to a fake hand. Second, when pointing to the real hand, there was no significant difference in pointing error between partial vision and blindfolding, indicating that, at least for unimpaired individuals, allowing partial vision does not give an “advantage” that improves performance on the test. This validates the strategy of partial vision, which in term helps with task automation, as we discuss below. Third, we applied OpenPoint to UE proprioceptive testing in persons with a stroke and found that individuals post stroke exhibited increased pointing error that was correlated with finger proprioceptive error measured with an independent, robotic assessment. We will now discuss these results, followed by the study’s limitations and future research directions.

### 4.1. Validating OpenPoint as a Proprioceptive Assessment

Finger-to-finger pointing without vision is sometimes used in neurological exams as a test of proprioception [32,61], but the results are qualitative. Here, we used recent advances in deep learning for computer vision to quantify finger pointing errors. We validated that pointing error measured for this task is indeed a sensitive test of UE proprioception in four ways.

First, when we removed proprioceptive input from the target hand by having participants point to a fake hand, pointing error increased. Theoretically, it is possible to point accurately to a fake hand by estimating the location of the fake fingers even if they are visually obscured. Participants were given a chance to view the fake hand before it was obscured, and, if they built a visual model of it (which would be similar to a visual model of their own hand), they theoretically should have been able to estimate how to point accurately to it. But this was not the case: error increased significantly when pointing to the fake hand. This demonstrates that the finger-to-finger pointing task depends on localization of the target hand as mediated by proprioceptive information from that hand. Therefore, proprioceptive impairment in the target hand should manifest as increases in pointing error above normative values. This means that the task fundamentally depends on proprioceptive information being available from the target hand. Moreover, the unimpaired motor system performed the pointing task equally well using vision plus proprioception or proprioception alone.

Second, consistent with this, OpenPoint detected increased proprioceptive pointing error in a group of persons with chronic stroke. A variety of studies using various assessment techniques have observed increased UE proprioceptive error after stroke [62,63]. The finding that OpenPoint also detected this phenomenon further validates its potential use in a clinical condition in which UE proprioceptive deficits are common.

Third, pointing error measured after stroke with OpenPoint moderately correlated with finger proprioception error measured with an independent technique, i.e., Crisscross applied with a robot [27,28,44,60]. This was somewhat surprising to us. Horvath et al. recently noted that proprioception assessments protocols, of which there are over 1300, seldom generalize to each other [38]. However, Horvath’s observation was focused on non-clinical populations. Damage to somatosensory processing brain areas might be expected to cause a generalized decrease in proprioceptive ability. Regardless, this result serves as a further validation of OpenPoint, which in this case demonstrates “concurrent validity” as recommended in measurement theory [64].

Fourth, pointing error increased when the target hand was held in an extended position away from the body. We found no impact of age on hand location proprioception error and found consistent orientation effects across ages. We tested this condition because Wilson et al. [56] found, using a robotic manipulandum, that proprioceptive errors increased when the hand was moved farther from the body. Confirming this result with the low-cost, OpenPoint system validates the idea that this approach has potential to be used in scientific studies of UE proprioception.

### 4.2. Validating the Partial Vision Testing Condition

For proprioceptive assessment, it is normal to blindfold the test subject or have them close their eyes. Asking subjects to close their eyes is susceptible to “cheating”, and blindfolding subjects requires an extra step that takes time and makes the subject feel vulnerable. Further, if we blindfolded subjects, then the OpenPoint visual interface would not be able to visually guide them through the assessment. Therefore, we tested whether obscuring the target hand with a computer-vision-generated polygon was equivalent to eliminating visual cues compared to a blindfolded condition. Pointing error was comparable in the two conditions, at least for unimpaired individuals pointing to their real hand, indicating that partial vision was equally effective as blindfolding in this condition.

However, when unimpaired individuals pointed to the fake hand, pointing error in the partial vision condition was significantly less than pointing error in the blindfolding condition. This suggests that the two visual conditions are not equivalent when the proprioceptive ability of the target hand is impaired. This is likely because the partial visual condition allows participants to improve estimates of location of the (fake) target finger compared to blindfolding. Nevertheless, pointing error still increased with partial vision for the fake hand compared to pointing to the real hand, indicating that proprioceptive information from the target hand is also used in the partial vision condition. Given these findings, it will be necessary to refer to the norms we have established here that are specific to the partial vision condition when assessing if there is impairment.

Another possibility besides relying on the partial vision of the screen would be to blank the screen after the participant points to the start position. We did not use this approach because we felt that keeping the screen active with a live video of the person helps hold their vision on the screen. Blanking the screen would likely make it more tempting for subjects to take a quick look down at their target hand, potentially confounding the results.

### 4.3. Effect of Age on Pointing Error

There was no significant difference in pointing error between the younger and older adult groups when they pointed to their real hand. This is consistent with previous research, where subjects reached reasonably accurately to the different targets of their own hand whether reaches were first made with vision or under proprioceptive guidance. Thus, the order in which Task 1, Task 2, and Task 3 were conducted is not strictly relevant.

Many studies have observed age-related deficits in proprioception [65,66] but others have not [67,68], including one study that found that younger and older adults display very good to excellent proprioceptive acuity in arm movement tasks [37]. Thus, although proprioceptive ability declines with age, these declines may not as readily impact proprioceptive pointing tasks involving both hands. In addition, our “older” group may simply not have been old enough to show an effect of age, since their mean age was 62.

For the younger group, we did not randomize the order of the tasks, so they always reached to the fake hand with full vision first, followed by the partial vision and blindfolded conditions. This may have influenced the results with respect to the effects of age. However, we showed that there were no significant differences in pointing error due to the task order. Therefore, this supports the finding of an effect of age rather than an effect of task order, i.e., the older participants had greater pointing error to the fake hand. Thus, they were more sensitive to the loss of proprioceptive information from the target hand compared to younger adults. One possible explanation is that the older adults were worse at visually estimating the location of the fake hand in the partial vision condition, or, for the blind-folded condition, in remembering the location of the fake hand. Another possibility is that they had proprioceptive loss in the pointing hand, but that this loss was masked when they could combine proprioceptive information from the two hands to guide pointing. A third possibility is that older adults rely more on vision than proprioception for upper extremity movements [69], and thus, when their vision was partially limited, it may have led to larger errors.

### 4.4. Making OpenPoint Feasible for Assessment of Proprioception in Stroke

In this study, we applied OpenPoint to people who had experienced a stroke. Proprioceptive impairments frequently occur following a stroke and are linked to diminished motor recovery as well as suboptimal rehabilitation outcomes [62,63]. Stroke commonly also causes UE weakness, and this must be considered in proprioceptive testing. For example, for the finger-to-finger pointing task, it is common to ask the subject to extend the arms in front of the body. This is not possible for many people with stroke, including many who participated here. Our solution was to modify the task so that participants held their target hand in front of their torso. This also served to position the target hand out of the participant’s field of view. All the participants with stroke could position the hand in front of their torso, but it was fatiguing for some, so we adopted the use of a pillow or a simple arm sling to alleviate fatigue, making the test feasible even for people with severe arm motor impairment with this simple modification.

Some of the individuals who participated also could not fully extend their fingers. We adapted the test to them by having them point to the most distal finger joint (PIP or MCP) that they were able to display. This was possible because HandMediaPipe tracks 21 points on the hand. This capability made the test feasible even for people with severe hand tone.

### 4.5. Limitations and Future Directions

There are several potential limitations of the approach presented here that suggest some directions for future research.

One limitation of this study is that we did not perform sample size estimation before starting the study. This was because the technological methodology was novel, and thus we had no previous data for such estimation. Instead, we used a convenience sample of a size comparable with several previous studies of computer vision-based assessments [41,49,55], proprioceptive testing [40,46,60,70], and proprioceptive pointing accuracy [71,72,73,74], with many in the range of 10–30 participants. The data presented here can be used for sample size estimation for future studies and will be made freely available upon reasonable request.

Another potential limitation of the study is that we allowed participants with poorer hand function after stroke to point to the PIP or MCP joints instead of the fingertips. While this makes the test accessible for individuals with different levels of impairment, previous research with unimpaired individuals suggests that proprioceptive errors when pointing to the knuckles or MCP joints are lower than when pointing to the fingertips, when vision is occluded [72,73]. Thus, we may have underestimated proprioceptive deficits for the individuals who pointed to PIP or MCP joints, compared to the age-matched controls who always pointed to their fingertips. However, even with this caveat, we found that the post-stroke group had increased proprioceptive error, further suggesting that this assessment can detect proprioceptive impairment. Moving forward with OpenPoint as an assessment technique for tracking the progression of proprioceptive deficits across time, it will be important to standardize the pointing targets for each subject across assessment sessions. Establishing proprioceptive error norms for different possible targets is also an important direction for future research.

Focusing on frontal-plane pointing error has a potential shortcoming, which is that frontal-plane error will be zero when the fingertips appear to be touching from the camera perspective but are separated in depth. If this “optical illusion” condition happens more frequently than other types of frontal-plane errors, it will seem like individuals have low proprioceptive error when they actually do not. However, two previous studies of a finger-to-finger pointing task indicate that errors are randomly distributed in the three cartesian directions for unimpaired individuals [73,74]. Thus, 2D frontal-plane errors should reflect proprioceptive ability in an unbiased way, at least for unimpaired individuals.

We tested the unimpaired individuals first, and we asked them to hold their hand close to their torso without touching it. Our goal was to eliminate the possibility that tactile information from the torso could be used to guide pointing. However, when we subsequently started testing people post stroke, it quickly became apparent that we needed to have them rest their hands against a cushion, to prevent fatigue. This may have had the side effect of decreasing their sagittal pointing error, since they now had a hard constraint that prevented errors “into” the body, and further. Moreover, they might have had increased information about their target hand location because of the tactile pressure it created against their torso. However, if use of the cushion decreased error, it did not decrease it enough to mask the increased pointing error post stroke, which was our key finding from the post-stroke data (as well as the correlation between pointing error and independent robotic measure of finger proprioception error). In using OpenPoint as an assessment tool going forward, it will be important to standardize the target hand position in a way that is appropriate for the test population. Resting the target hand against a cushion on the torso seems like a logical approach post stroke, but future research should also determine the extent to which torso tactile information might help guide pointing.

The accuracy of our assessment is influenced by the limitations of the MediaPipe Hands model [51], which can be affected by factors like camera resolution, lighting conditions, distance from the camera, motion speed, and viewing angle [75]. To ensure optimal results, we controlled these variables by using an HD camera (1080 p × 720 p) in a room with controlled lighting and no reflections, with the camera centered and at a close distance from the participant and controlling that the participant moved at medium speeds. The model is built to be robust, and it can handle a broad range of standard camera resolutions from smartphones and webcams. Some similarities have been found between MediaPipe and standard methods for tracking upper extremity movements [76] (preprint), but future work should focus on improving 3D accuracy using 2D image data to enhance MediaPipe’s applicability in clinical populations. For instance, [77] demonstrated that pose estimation models generally exhibited strong and significant correlations with 3D motion analysis for most activities. This finding suggests that image-based models could be effectively utilized in remote rehabilitation settings.

Despite the instruction to stop moving the finger after their initial pointing movement, some participants attempt to correct their movement to perfectly align the fingertips. In such instances, we manually identified the frame prior to movement correction. Automating this process to control for corrective movements is a challenge for future research.

MediaPipe returns marker locations in pixel coordinates, but we wanted a measurement in real-world conditions. We obtained images of each stroke participant’s hand on graph paper to calibrate pointing errors. However, for the unimpaired participants, we did not collect calibration pictures. Instead, we utilized the average hand sizes derived from the stroke participants’ photos on a sex-related basis. This approach likely introduced some variability in the pointing error estimates for the unimpaired participants. In future work, we plan to automate hand size calculation.

Another potential limitation is that OpenPoint focuses on a single method to test proprioception. As mentioned above, a recent review of proprioception testing protocols found over 1300 and suggested that they rarely generalize. In the present study, we found that OpenPoint pointing error did generalize to a robotic measurement of finger proprioception (Crisscross) after stroke. This was somewhat surprising to us, as OpenPoint measures whole arm proprioception (including the finger) and Crisscross measures only distal, finger proprioception. Further, OpenPoint is an active proprioception test, requiring voluntary movement of the pointing hand, while Crisscross is purely passive. As mentioned above, this generalization may be due to stroke causing broad damage in somatosensory brain areas. Nevertheless, it is possible to automate other types of tests of proprioception using computer vision, which might also hold value. What the minimal viable set of proprioception tests is remains unknown.

We tested OpenPoint with persons who were older and persons who had experienced a stroke, both conditions known to affect proprioception. Future research should investigate if it holds value as a proprioception assessment in other conditions known to affect proprioception, of which there is a wide variety.

Finally, we used a desktop computer and webcam in this study. OpenPoint could also be implemented using a smartphone, a direction we are pursuing. We have already developed and released an app called Proprio that uses a similar approach to OpenPoint to gamify finger-to-finger proprioception training [78]. In Proprio, players play a musical game by pointing to different fingers on their target hand to play notes, repeatedly challenging their proprioceptive system. Using markerless motion tracking to produce practical ways to retrain proprioception is an interesting direction for additional research.

Our hope is that this technology helps make quantitative proprioception testing more widely available, improving outcomes for people with proprioceptive deficits.

## 5. Conclusions

We leveraged recent advances in deep-learning-based computer vision to automate an established neurologic test of UE proprioception. We validated OpenPoint with young, older, and post-stroke participants. We believe this approach can provide the basis for assessing finger proprioception quickly, simply, and inexpensively in clinical settings, or, at home, for telerehabilitation/telemedicine applications.

## Data Availability

The original OpenPoint code is openly available in GitHub at https://github.com/gcornella/OpenPoint (accessed on 8 October 2024). Datasets generated and/or analyzed during our work are made available upon reasonable request.
